# Anti-Inflammatory Effects of *Phlebia* sp. Extract in Lipopolysaccharide-Stimulated RAW 264.7 Macrophages

**DOI:** 10.1155/2022/2717196

**Published:** 2022-07-14

**Authors:** Eui Hyeon Lim, Seul-Ki Mun, Jong-Jin Kim, Dong-Jo Chang, Sung-Tae Yee

**Affiliations:** ^1^Department of Pharmacy, Sunchon National University, 255 Jungang-Ro, Suncheon 57922, Republic of Korea; ^2^Department of Biology, Sunchon National University, 255 Jungang-Ro, Suncheon 57922, Republic of Korea

## Abstract

Lichens are a life form in which algae and fungi have a symbiotic relationship and have various biological activities, including anti-inflammatory and antiproliferative activities. This is the first study to investigate the anti-inflammatory activity of a *Phlebia* sp. fungal extract (PSE) isolated from *Peltigera neopolydactyla* in lipopolysaccharide- (LPS-) stimulated RAW 264.7 macrophage. PSE reduced the production of the proinflammatory cytokine (tumor necrosis factor-*α*, interleukin-6, and interleukin-1*β*), chemokine (granulocyte-macrophage colony-stimulating factor), nitric oxide, and prostaglandin E2 in the LPS-stimulated RAW264.7 macrophages. Especially, PSE inhibits the phosphorylation of activator protein-1 (AP-1) signaling (c-Fos and c-Jun) and their upstream mitogen-activated protein kinase kinases/mitogen-activated protein kinases (MKK/MAPKs: MKK4, MKK7, and JNK) and finally reduced the production of the inflammatory cytokines. The inhibitory effects mainly act via suppressing JNK-mediated AP-1 rather than the NF-*κ*B pathway. Furthermore, PSE inhibited the production of final inflammatory effector molecules involved in AP-1 signaling, including nitric oxide (NO) and prostaglandin E2 (PGE2). Here, we report that PSE has the potential to be developed as an anti-inflammatory agent.

## 1. Introduction

Macrophages play a critical role in the immune system by serving as the first line of defense against pathogens and tumors and also link innate and adaptive immunity [1]. However, excessively activated macrophages release various inflammatory mediators such as nitric oxide (NO) and cytokines and prostaglandin E2 (PGE2) leading to uncontrolled inflammation [2]. The aforementioned inflammatory factors are regulated by several signaling pathways such as up mitogen-activated protein kinase kinases/mitogen-activated protein kinases (MKK/MAPKs), nuclear factor kappa B (NF-*κ*B), and activator protein-1 (AP-1) [3]. Abnormal phosphorylation of inflammation factors induces destructive situations and has been found to cause severe tissue damage, endotoxin shock, and chronic inflammatory response, leading to a series of diseases such as cancer [4, 5]. Therefore, inhibition of NF-*κ*B or AP-1 activation is considered an important strategy for treatment of inflammatory diseases [3, 5].

Lichens are complex organisms composed of fungi and photosynthetic partners (algae or cyanobacteria) [6]. Lichens are characterized by their sensitivity to environmental stresses, such as pollution and climatic changes [7]. Moreover, lichens have been used as food ingredients and herbal tea in Japan and China [1]. Lichens produce secondary metabolites that have a variety of biological activities, such as anti-inflammatory, antitumor, antioxidant, and immune-suppressive activities [1, 8, 9]. *Phlebia* sp. is an endolichenic fungi constituting the lichen, *Peltigera neopolydactyla*, and belongs to the family Meruliaceae and the order Polyporales [10].

There have been no reports of the anti-inflammatory effects of *Phlebia* sp. extracts (PSE). Herein, we report that PSE inhibits AP-1 signaling on the activated-macrophage by regulating mitogen-activated protein kinase kinases (MMK) and mitogen-activated protein kinases (MAPKs). Effector molecules in downstream of AP-1 signaling, including proinflammatory cytokines (TNF-*α*, IL-1*β*, IL-6, and GM-CSF), NO, and PGE2, are decreased by treatment of PSE on LPS-stimulated RAW 264.7 cells. Based on our results, we conclude that PSE can be used for the development of material and functional products for anti-inflammation.

## 2. Materials and Methods

### 2.1. Sample Collection, Identification, and Extraction

*Peltigera neopolydactyla* was collected from the coastal rocks of southern Korea in 2018 and deposited at the Korean Lichen and Allied Bioresource Center (KOLABIC) of the Korean Lichen Research Institute (KoLRI), Sunchon National University, Korea. For the accurate classification of the fungal part of the sample, the ribosomal DNA internal-transcribed spacer region (ITS) was analyzed to confirm that it was *Phlebia* sp. To obtain the ethyl acetate (EA) PSE, *Phlebia* sp. was cultured on malt extract agar. Then, three to four fungal agar pieces were obtained and placed in 200 mL of malt and yeast extract broth (MY) and incubated for one month with shaking at a 150 rpm at 15°C. After adding an equivalent amount of EA to the culture for 2 h, the culture was thoroughly mixed and filtered using 3 M filter paper, and the water/EA layer was separated. The EA layer obtained through this process was evaporated using a vacuum rotary evaporator, and 5 mL of EA was added to the evaporated product. The product was subsequently sonicated, and the EA was evaporated in an oven. The product was then stored as powder in samples vials.

### 2.2. Chemicals and Reagents

Fetal bovine serum (FBS) and Roswell Park Memorial Institute 1640 medium (RPMI 1640) were purchased from Hyclone Laboratories (Hyclone, South Logan, UT, USA). Dimethyl sulfoxide (DMSO), 2-mercaptoehanol, and lipopolysaccharides (*Escherichia coli* 0111:B4) were purchased from Sigma-Aldrich (St. Louis, MO, USA). The Cell Counting Kit-8 (CCK-8) was purchased from Dojindo Laboratories (Dojindo, Kumamoto, Japan). Purified hamster anti-mouse (TNF-*α*), purified rat anti-mouse (IL-6, GM-CSF), biotin human anti-mouse (TNF-*α*), and biotin rat anti-mouse (IL-6, GM-CSF) were purchased from BD Biosciences (San Jose, CA, USA). The IL-1*β* ELISA kit was acquired from Thermo Fisher (Rockford, IL, USA). The PGE2 ELISA kit was purchased from R&D Systems (Minneapolis, MN, USA).

### 2.3. Cell Culture

RAW 264.7 (murine macrophage cell line, KCLB 40071) cell line was acquired from the Korean Cell Line Bank (Seoul, South Korea). The cells were cultured in RPMI 1640 supplemented with 10% fetal bovine serum, 100 units/mL penicillin, 100 *μ*g/mL streptomycin (Invitrogen, Carlsbad, CA, USA), and 2-mercaptoethanol (50 *μ*M), in a humidified atmosphere at 37°C with 5% CO_2_.

### 2.4. Cytotoxicity Assay and NO Production

RAW 264.7 cells (5 × 10^4^ cells/well) were seeded in 96-well plates and incubated overnight. The next day, each well was preincubated with LPS (1 *μ*g/mL) for 1 h and treated with 1, 3, 10, 30, and 50 *μ*g/mL of the PSE for 24 h at 5% CO_2_ and 37°C. Then, the half of supernatant was removed from each well, and CCK-8 (10 *μ*L) was added to the wells. The optical density was measured at 450 nm using a microplate reader (Versa Max, Molecular Devices, Sunnyvale, CA). The supernatant was used to measure the NO production, using the Griess assay [1].

### 2.5. Cytokine Assay

RAW 264.7 cells (5 × 10^5^ cells/well) were seeded in 12-well plates and incubated overnight. The next day, each well was preincubated with LPS (1 *μ*g/mL) for 1 h and treated with 1, 3, 10, 30, and 50 *μ*g/mL PSE, for 24 h at 5% CO_2_ and 37°C. Cytokine concentrations were determined by using ELISA.

### 2.6. Western Blot Assay

RAW 264.7 cells were incubated overnight in a 6-well plate at a density of 1 × 10^6^ cells/well. Then, PSE was added to the cells and incubated for 4 h or 18 h. The cells were washed once with cold phosphate-buffered saline (PBS) on ice and lysed in radioimmunoprecipitation assay (RIPA) buffer containing a phosphatase and protease inhibitor cocktail (Thermo, Rockford, IL, USA). Proteins were obtained by centrifugation at 14,000 × g and 4°C for 20 min. The concentration of the protein samples was determined using a bicinchoninic acid (BCA) protein assay kit (Thermo, Rockford, IL, USA). Protein samples were separated using 4-12% bis-tris plus gels (Thermo, Rockford, IL, USA) and transferred to nitrocellulose membranes (Thermo, Rockford, IL, USA). The membranes were incubated with blocking solution for 3 h and then incubated overnight at 4°C with primary antibodies. The primary antibodies included *β*-actin (1 : 2000, Thermo, Rockford, IL, USA), inducible NO synthase (iNOS), cyclooxygenase-2 (COX-2), c-Jun, p-c-Jun, c-Fos, p-c-Fos, p38,p-p38, ERK, p-ERK, JNK, p-JNK, MKK4, p-MKK4, MKK7, p-MKK7, I*κ*B, p-I*κ*B, IKK, p-IKK, NF-*κ*B (p65), and p-NF-*κ*B (p-p65). All primary antibodies for Western blot were purchased from cell signaling technology except *β*-actin. The membranes were washed with TBST (Thermo, Rockford, IL, USA) and incubated with horseradish peroxidase- (HRP-) conjugated secondary antibody (Thermo, Rockford, IL, USA) for 3 h at room temperature with shaking. Membranes were treated with an enhanced chemiluminescence kit (Thermo, Rockford, IL, USA). Protein bands were captured using the ChemiDoc Imaging System (Bio-Rad, Hercules, CA, USA).

### 2.7. Immunofluorescence Staining

RAW 264.7 cells were cultured 24 h in a 96-well plate (165305, Thermo) at a density of 5 × 10^4^ cells/well. Cells were preincubated with LPS (1 *μ*g/mL) for 1 h and treated with 50 *μ*g/mL PSE, for 24 h at 5% CO_2_ and 37°C. Briefly, cells were fixed and permeabilized using intracellular staining kit (130-093-142, MACS). Cells were incubated with an anti-p65 primary antibody (3031, Cell signaling) at 4°C 30 min, and plate were washed twice with PBS and incubated with anti-rabbit IgG Alexa488-conjugated secondary antibody (A11034, Invitrogen) which was added for 1 h. After washing with PBS and staining with Hoechst 34580 (H21486, Thermo), cells were imaged by fluorescent microscope (EVOS M7000, Thermo).

### 2.8. Statistical Analysis

Data are presented as mean ± standard deviation (SD). Statistical differences between groups were analyzed using one-way analysis of variance (ANOVA) followed by Duncan's test using SPSS version 28 (Chicago, IL, USA). A value of *p* < 0.05 was considered statistically significant.

## 3. Results

### 3.1. Viability and NO Inhibition Effect of PSE in RAW264.7 Macrophages

**Figure 1:**
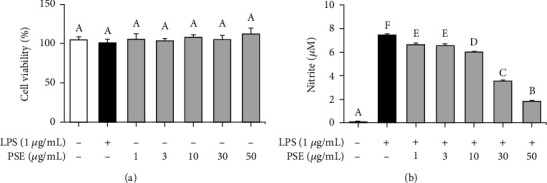
Confirmation of viability and NO inhibition effect of PSE in RAW264.7 macrophages. The cells were treated with different concentration of PSE for 24H and the viability measured by cell counting kit-8 assay (a). The cells were pretreated with 1 *μ*g/mL LPS for 1 h and subsequently treated with 1-50 *μ*g/mL PSE for 24 h. The concentration of nitric oxide in the culture medium was measured by using the Griess reaction (b). *p* value < 0.05 was considered statistically significant by Duncan's test.

Nitric oxide (NO) is a mediator and regulator of the inflammatory response and has many biological functions, including the elimination of bacteria and mediation of cell signaling [11, 12]. First, we have checked the cytotoxicity of PSE and the effect on NO production. Specifically, the anti-inflammatory activity of PSE was investigated in LPS-stimulated RAW 264.7 macrophages. The RAW 264.7 cells were exposed to varying concentrations of PSE, and the cytotoxicity was measured using CCK-8 after 24 h (Figure 1(a)). As shown in Figure 1(a), PSE has no cytotoxicity within treated concentrations to RAW 264.7 macrophages. In addition, the noncytotoxic PSE has an inhibitory effect on the NO production from LPS-stimulated RAW 264.7 macrophages in concentration-dependent manner (Figure 1(b)).

### 3.2. Inhibitory Effect of PSE on the Production of Inflammatory Cytokines and PGE2

**Figure 2:**
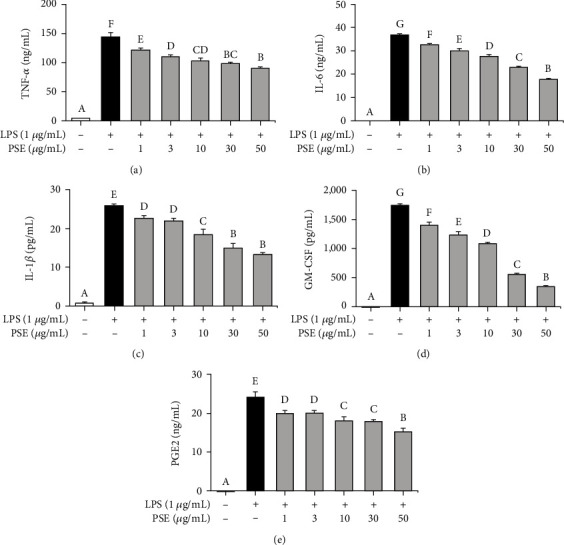
Inhibitory effect of PSE extract on the production of cytokines and PGE2. Effect of PSE on the proinflammatory cytokines and prostaglandin E2 (PGE2) produced by LPS-stimulated RAW 264.7 cells. The cells were pretreated 1 *μ*g/mL LPS for 1 h and treated with 1-50 *μ*g/mL PSE for 24 h. The culture supernatant was measured by ELISA (a–e). *p* value < 0.05 was considered statistically significant by Duncan's test.

Inflammatory cytokines, such as TNF-*α*, IL-6, and IL-1*β*, play an essential role in regulating inflammation [13]. GM-CSF regulates the phagocytosis of microbial pathogens by the activation of macrophages. Furthermore, GM-CSF induces differentiation and recruitment of inflammatory cells from the bone marrow into peripheral tissue. The aforementioned cytokines elicit an immune response and, simultaneously, uncontrolled immune response exacerbates inflammation, implying regulation of inflammatory cytokines is essentially required for the control of inflammation [14]. To investigate the levels of proinflammatory cytokines (TNF-*α*, IL-6, and IL-1*β*), macrophages were stimulated with LPS and treated with 1, 3, 10, 30, and 50 *μ*g/mL of PSE. As presented in Figures 2(a)–2(c), the PSE treatment inhibited cytokine productions in the LPS-stimulated RAW 264.7 macrophages.  Figure 2(d) shows a significant decrease in GM-CSF production by treating PSE. Furthermore, the PSE treatment inhibited the production of PGE2 in a concentration-dependent manner as shown in Figure 2(e). The biosynthesis of PGE2, which is regulated by COX-2, is increased significantly in inflammatory tissues, where it contributes to the occurrence of acute inflammation [12]. These results clearly indicate that PSE anti-inflammatory effects by regulating inflammatory cytokines and PGE2.

### 3.3. Inhibitory Effect of PSE on the Expression of iNOS and COX-2

**Figure 3:**
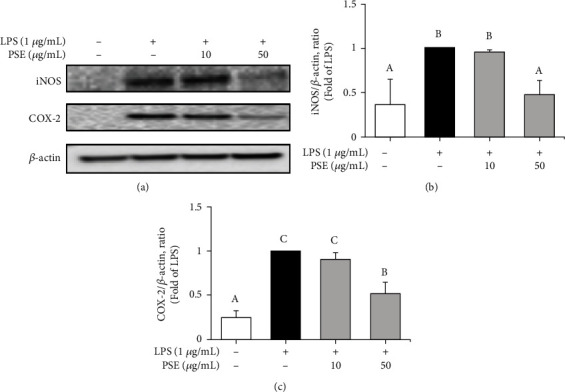
Inhibitory effect of PSE on the expression of iNOS and COX-2. The cells were pretreated 1 *μ*g/mL LPS for 1 h and treated with 10 and 50 *μ*g/mL PSE for 17 h. Western blot analysis was performed to confirm the effect of PSE on the expression of proinflammatory proteins (a). Ratios of iNOS (b) and COX-2 (c) are presented as the mean ± SD from three independent experiments. *p* value < 0.05 was considered statistically significant by Duncan's test.

In the inflammatory reaction, NO secretion is regulated by iNOS in macrophages, and the generated NO molecules work as an inflammatory mediator in immunity [12]. COX-2 is another enzyme that plays a pivotal role in the mediation of inflammation and catalyzes the rate-limiting step in prostaglandin (PG) biosynthesis [14]. To check the anti-inflammatory mechanism of PSE, we conducted Western blotting to confirm the expression of iNOS and COX-2. We observed a significant increase in the expression of iNOS and COX-2 by treating LPS on the RAW 264.7 macrophage, whereas the treatment of PSE suppresses the expression of iNOS and COX-2 concentration dependently (Figures 3(a)–3(c)).

### 3.4. Effect of the PSE on the NF-*κ*B Signaling Pathway

**Figure 4:**
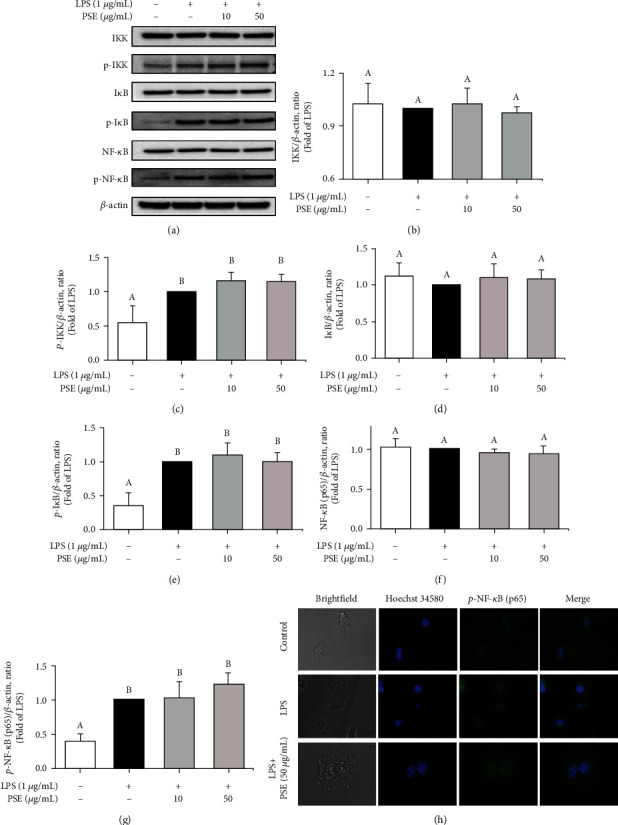
Effect of PSE on the activation of NF-*κ*B signaling. The cells were pretreated 1 *μ*g/mL LPS for 1 h and treated with 10 and 50 *μ*g/mL PSE for 3 h. Expression of IKK, p-IKK, I*κ*B, p-I*κ*B, NF-*κ*B, and p-NF-*κ*B were determined by Western blot analysis (a). The ratios of IKK (b), p-IKK (c), I*κ*B (d), p-I*κ*B (e), NF-*κ*B (f), and p-NF-*κ*B (g) are shown. The cells were pretreated 1 *μ*g/mL LPS for 1 h and treated with 50 *μ*g/mL PSE for 24 h. The localization of phosphor-NF-*κ*B (p-p65) and nuclei were determined by staining with anti-phospho-p65 (green) and Hoechst 34580 (blue) (h). Images were obtained by microscopy at 40x magnification. Data are presented as mean ± SD from three independent experiments. *p* value < 0.05 was considered statistically significant by Duncan's test.

Various molecules in signal transduction pathways with complicated networks affect inflammation. To investigate the effect of PSE on the NF-*κ*B signaling pathway, LPS-stimulated RAW 264.7 macrophages were treated with PSE to analyze the expression of inflammatory mediators by Western blots. The results showed that PSE could not inhibit the levels of p-IKK, p-I*κ*B, and p-NF-*κ*B induced by LPS (Figures 4(a)–4(f)). In addition, immunofluorescence results showed that 50 *μ*g/mL of PSE did not inhibit the nuclear potential p-NF-*κ*B induced by LPS (Figure 4(h)). These results suggested that PSE did not block the NF-*κ*B signaling pathway.

### 3.5. Anti-Inflammatory Activity of the PSE through Inhibition of AP-1 Pathway

**Figure 5:**
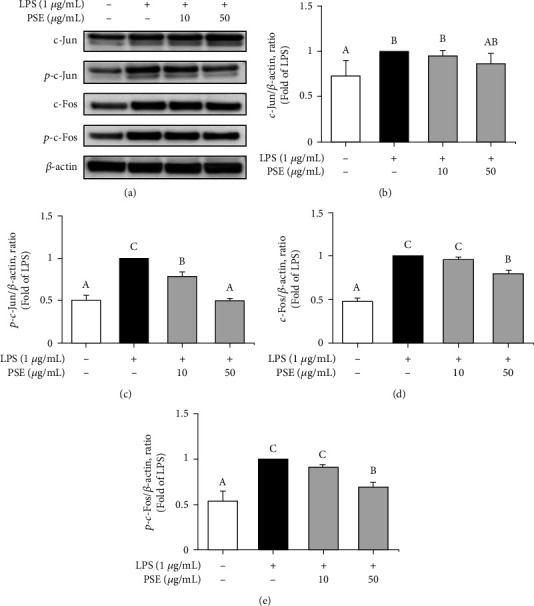
Inhibitory effect of PSE on the activation of AP-1 signaling. The cells were pretreated 1 *μ*g/mL LPS for 1 h and treated with 10 and 50 *μ*g/mL PSE for 3 h. Expression of c-Jun, p-c-Jun, c-Fos, and p-c-Fos were determined by Western blot analysis (a). The ratios of c-Jun (b), p-c-Jun (c), c-Fos (d), and p-c-Fos (e) are shown. Data are presented as mean ± SD from three independent experiments. *p* value < 0.05 was considered statistically significant by Duncan's test.

AP-1 is a pivotal factor in the regulation of inflammation by producing proinflammatory mediators and cytokines such as iNOS, COX-2, IL-1*β*, and IL-6 [15]. Phosphorylation of c-Jun, which is a member of the AP-1 family, induces translation of gene, and produces protein for TNF-*α* [16]. c-Fos is one of the most powerful transcription factors in the immune system and belongs to the Fos family. c-Fos binds to c-Jun to form active AP-1 and plays a regulatory role in inflammation [17]. LPS-induced RAW 264.7 macrophages were treated with 10 and 50 *μ*g/mL of PSE to confirm the effect on c-Jun and c-Fos, and their expressions were measured by Western blot assay (Figure 5(a)). Figures 5(b)–5(e) shows the quantitative analysis data with Figure 5(a) for c-Jun and c-Fos after-treatment of the RAW264.7 macrophages with PSE. These results clearly illustrate that treatment of the macrophages with PSE suppressed the production of inflammatory proteins, which is phosphorylation of c-Jun, c-Fos, and total c-Fos.

### 3.6. Anti-Inflammatory Activity of the PSE on the MKK/MAPK Pathway

**Figure 6:**
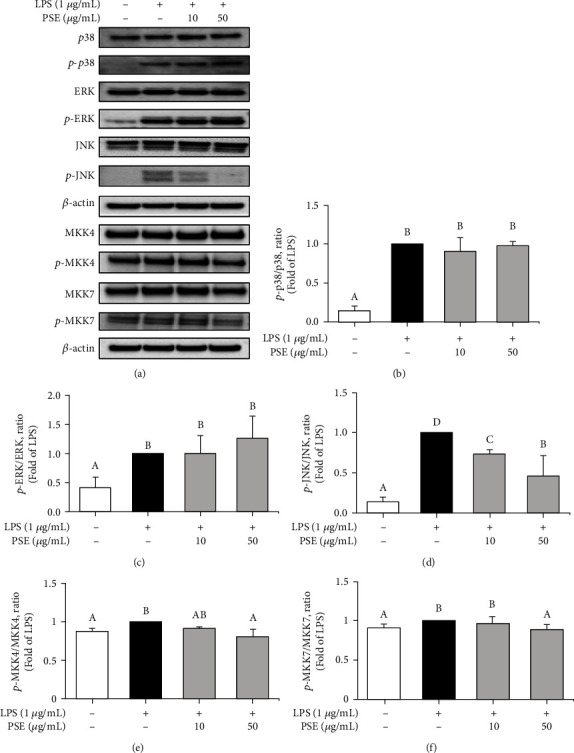
Inhibitory Effect of PSE on the activation of MKK/MAPK signaling. Expression of MKK/MAPK signaling was determined by Western blot analysis (a). The cells were pretreated 1 *μ*g/mL LPS for 1 h and treated with 10 and 50 *μ*g/mL PSE for 3 h (b–d). The cells were pretreated 10 and 50 *μ*g/mL PSE for 30 min and treated 1 *μ*g/mL LPS for 60 min (e, f). Data are presented as mean ± SD from three independent experiments. *p* value < 0.05 was considered statistically significant by Duncan's test.

Activated MAPKs phosphorylate several transcription factors, such as c-Jun and c-Fos [18], and c-Jun kinase (JNK), a member of MAPKs, is a key player in inflammation as stress-activated protein kinase [19]. Numerous reports, including increase of inflammatory cytokines production by MAPKs (ERK, p38, and JNK), have demonstrated that MAPKs are important targets for inflammation regulation [19, 20]. We have first investigated the inhibitory effect of the PSE on the phosphorylation of p38 and ERK, but it did not affect it (Figures 6(b) and 6(c)). However, phosphorylation of JNK was induced by LPS which was increased, but PSE inhibits the phosphorylation of JNK concentration dependently (Figure 6(d)). JNKs are activated through the sequential activation of protein kinases containing two dual-specificity MAP kinase kinases (MKK4 and MKK7) [21, 22]. We measured the effect of PSE on the phosphorylation of MKK4 and MKK7 (Figures 6(e) and 6(f)), showing the phosphorylation of MKK4 and MKK7 was reduced by PSE treatment. Taken together, PSE inhibits phosphorylation of MKK4 and MKK7, which results in blocking the production of inflammatory cytokines and effector molecules via the decrease of phosphorylation of JNK.

## 4. Discussion

Inflammation occurs by fighting for the eradication of invading bacteria. Toll-like receptor 4 (TLR4) belongs to the TLR family of receptors that induce a proinflammation response to pathogens like LPS [23]. Activation of TLR4 and CD14 receptors plays a critical role in the induction of inflammatory processes. Stimulating TLR4 induces activating various adaptor proteins, such as TIRAP and MyD88, and consecutively induces activation of NF-*κ*B and AP-1, leading to the transcription of inflammatory genes [24]. Excessive inflammation exacerbates pathological symptoms in various diseases including asthma, diabetes mellitus, inflammatory bowel disease, obesity, and sepsis [25]. Therefore, it is important to regulate inflammation for understanding inflammatory diseases and developing medications, and one of potential strategies for regulation of inflammation is to inhibit the signaling pathways of transcription factors such as NF-*κ*B and AP-1 [26].

Lichen is a symbiotic cyanobiont, photobiont, and mycobiont [27]. Lichen extracts have the ability for antioxidant, antiviral, antitumor, and anti-inflammatory activities under experimental conditions [28]. Endolichenic fungi are ubiquitous in the environment and have been investigated for their antimicrobial, antineuroinflammatory, and anticancer effects [29–32].

We have investigated the anti-inflammatory effects of PSE by using LPS-activated macrophages to confirm phenotypic changes and intracellular signal transduction. Inflammation is regulated by expression levels of iNOS and COX-2 and produces large amounts of the inflammatory effector molecules, NO, PGE2, and cytokines [33]. Increased inflammatory cytokines make synergic effects exacerbate inflammation. TNF-*α* is critical for the synergistic induction of NO synthesis in macrophages. In addition, IL-1*β* and IL-6 are believed to be endogenous mediators of LPS-induced fever [7, 33], GM-CSF also upregulates the expression of TLR2, TLR4, and CD14 [14]. Here, we confirmed that PSE significantly inhibited the production of inflammatory cytokines including TNF-*α*, IL-1*β*, IL-6, and GM-CSF. However, PSE did not inhibit the phosphorylation of I*κ*B by stimulating LPS and subsequently did induce the phosphorylation and translocation of NF-*κ*B.

**Figure 7:**
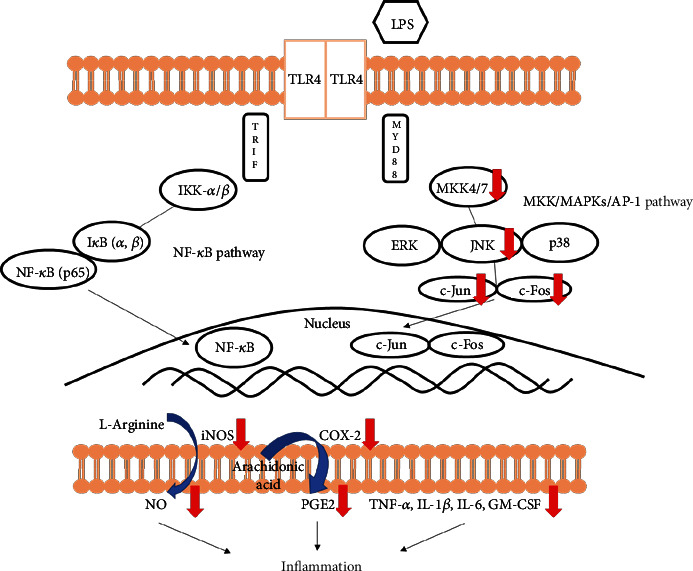
Schematic representation of the anti-inflammatory effects of *Phlebia* sp. extract in RAW 264.7 macrophages.

Interestingly, we observed the activated macrophages by LPS induce increased phosphorylation of MAPK family proteins whereas PSE inhibits MAPKs, particularly JNK, and upstream pathway, MMK4/7. Reduced MAPK signaling leads to downregulation of the AP-1 pathway by the decrease of phosphorylated c-Jun and c-Fos. Resultingly, inflammatory effector molecules NO and PGE2 correlate with expression patterns of the signaling pathways in company with reduced inflammatory cytokine levels (Figure 7). Based on these results, PSE can be a candidate for specific inflammation regulators by inhibiting particular intracellular pathways of inflammation.

## 5. Conclusion

PSE inhibits the production of NO, PGE2, and proinflammation cytokines, such as TNF-*α*, IL-6, IL-1*β*, and GM-CSF in LPS-stimulated macrophages by downregulating expression of iNOS and COX-2. Those phenomena are affected by inhibitory effects of PSE at the phosphorylation of MKK4/7, JNK, and AP-1 pathways. Collectively, these results demonstrate that PSE has therapeutic potential and can be developed into a novel anti-inflammatory agent.

## Data Availability

The raw data, supporting the conclusions of this manuscript, are available by contacting the corresponding author.
